# Cerebral Sinus Thrombosis and Immune Thrombocytopenia Post COVID-19 Vaccination: A Case Report and Narrative Review

**DOI:** 10.7759/cureus.34550

**Published:** 2023-02-02

**Authors:** Azka Ali, Arthur Dilibe, Shilpa Rai, Ayoola Awosika, Adekunle E Omole, Maryam Ahmed, Stella Nwosu

**Affiliations:** 1 Internal Medicine, Rosalind Franklin University of Medicine and Sciences, Chicago, USA; 2 Internal Medicine, East Carolina University Health, Greenville, USA; 3 Internal Medicine, Isra University, Hyderabad, PAK; 4 College of Medicine, University of Illinois, Chicago, USA; 5 College of Health Sciences and Professions, Ohio University, Athens, USA; 6 Anatomical Sciences, American University of Antigua, College of Medicine, Saint John, ATG; 7 Internal Medicine, Allied Hospital, Maple Heights, USA; 8 Hematology and Medical Oncology, Emory University, Atlanta, USA

**Keywords:** vaccine-induced prothrombotic immune thrombocytopenia (vipit), 2019 novel coronavirus, post-covid 19 vaccine complications, thrombosis with thrombocytopenia syndrome (tts), vitt covid-19

## Abstract

Vaccine-induced immune thrombocytopenia and thrombosis (VITT) following the adenoviral vector COVID-19 vaccine is a rare adverse event. Although the risk of VITT following the COVID-19 vaccine appears to be low, early diagnosis and management can be lifesaving. We present a case of VITT in a young female who presented with persistent headaches and fevers followed by anisocoria and right-sided hemiplegia. Initial imaging was unremarkable, and labs showed thrombocytopenia and elevated d-dimers. Repeat imaging revealed thrombosis in the left transverse and superior sagittal sinuses, and she was diagnosed with VITT. She received combined treatment with intravenous immunoglobulins and systemic anticoagulation, resulting in an increased platelet count and resolution of her neurological symptoms.

## Introduction

Vaccine-induced immune thrombocytopenia and thrombosis (VITT), also known as vaccine-induced prothrombotic immune thrombocytopenia (VIPIT), or thrombosis with thrombocytopenia syndrome (TTS) is a prothrombotic syndrome encountered in the recipients of vaccines such as measles, mumps, rubella, human papillomavirus, poliovirus, and hepatitis B virus [1]. VITT is a rare but life-threatening complication with high morbidity and mortality, and the estimated risk for TTS is at least 1:50,000 in patients below 50 years and 1:100,000 in the population above 50 years of age [2]. However, an increased risk has been reported in younger patients and the recipients of the first dose of the vaccine. VITT has a broad range of clinical manifestations depending on the involved vessel, such as a deep vein, acute arterial thrombosis, pulmonary embolism, cerebral, and splanchnic veins. In cases of cerebral involvement, VITT may manifest with sudden onset worsening headache, vomiting, and nausea followed by altered sensorium and neurological manifestations [3]. Although rare, VITT following coronavirus disease 2019 (COVID-19) vaccination has also been reported with high morbidity and mortality [4-6]. We report a case of VITT following the second dose of the COVID-19 vaccine.

## Case presentation

A 25-year-old female without any previous medical history was brought to the emergency department with complaints of fever, generalized headache, and blurry vision ongoing for the last two days. The fever was high-grade, associated with rigors and chills. She had developed flu-like symptoms after the second dose of AstraZeneca COVID-19 vaccine, five days prior to presentation. She received her treatment from the local physician and was treated with a provisional diagnosis of meningism.

On examination, she was anxious looking, hemodynamically stable, well oriented to time, place, and person with a temperature of 101^o^F. Her neurological examination was intact, and her initial laboratory results showed thrombocytopenia, elevated d-dimers, and c-reactive protein (Table 1).

**Table 1:** The results of initial laboratory tests.

Parameter	Result	Reference range
Red cell count	4.22	4.20-5.65 million cells/uL
White cell count	5.2	4000-11000/mm^3^
Platelet count	24,000	150,000-350,000/mm^3^
Hemoglobin	11.1	12.1-15.1 g/L
D-dimer	> 5000	< 0.50 ng/dl
Fibrinogen	1.6	1.8-3.6 g/L
Lactate dehydrogenase	279	140-289 IU/L
C-reactive protein	108	8-10 mg/L

Her COVID-19 polymerase chain reaction (PCR) was negative. Her chest x-ray was unremarkable, and her blood culture was negative. Brain computed tomography (CT) was negative for any acute abnormality, and a lumbar puncture was not performed due to thrombocytopenia. Her lab results were attributed to vaccination and managed symptomatically.

The following day, she presented again with persistent headaches, three episodes of projectile vomiting, anisocoria, and right-sided hemiplegia. There were no signs of meningeal irritation. On neurological examination, she had decreased power, grip, and sensations on the right side of the body. She had no previous medical history or family history of the thrombotic event. She denied any history of smoking, alcohol, or illicit drug use and was not taking any oral contraceptives or hormone replacement therapy.

**Figure 1:**
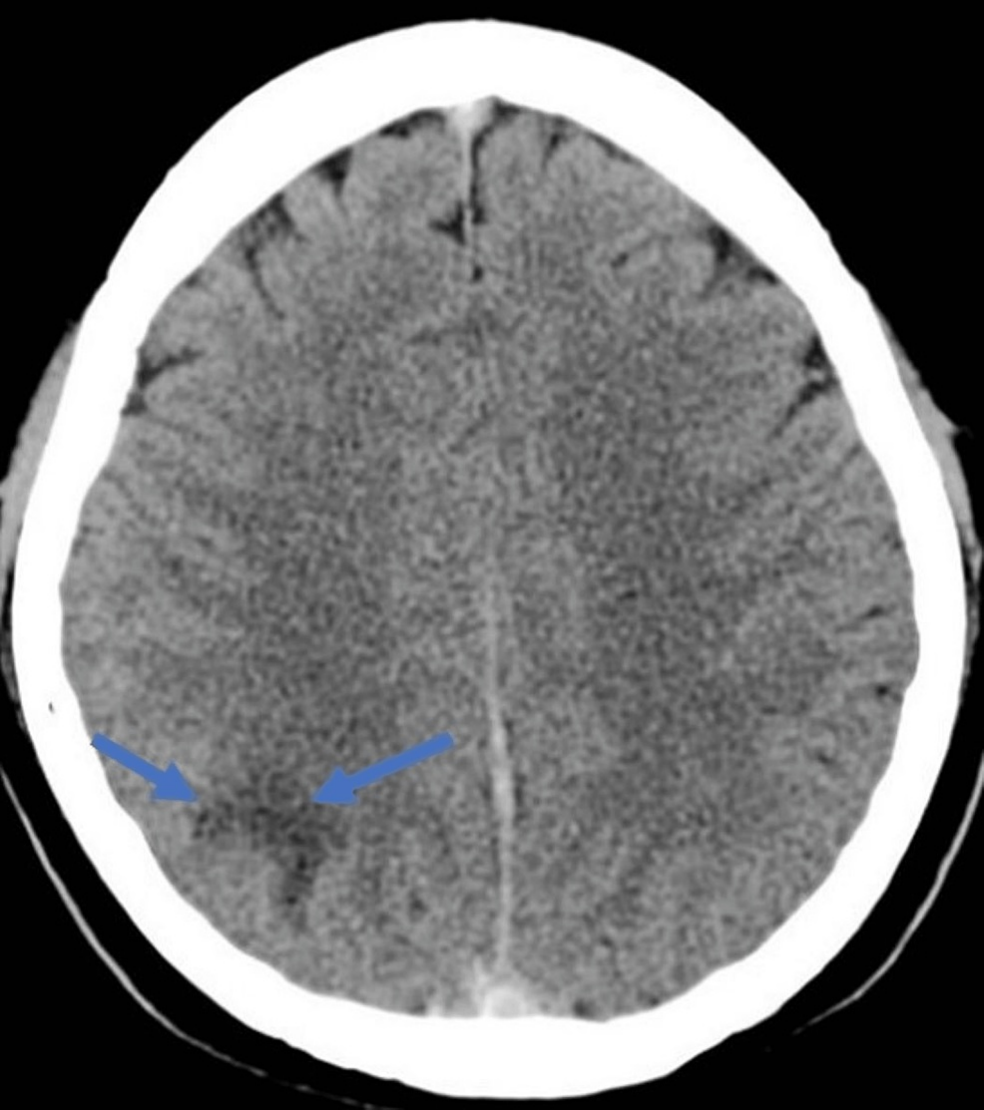
Computed tomography showing a region of low density of hemorrhagic focus in the right parietal lobe.

**Figure 2:**
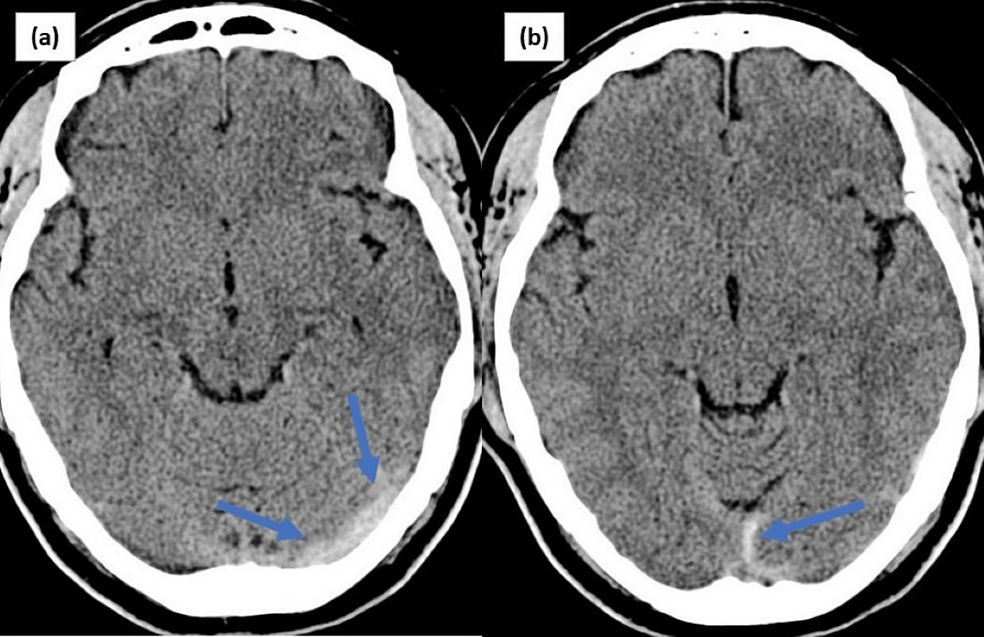
CT brain demonstrating hyperdense area showing thrombosis of left transverse sinus (a) and superior sagittal sinus (b).

She underwent a repeat brain CT (T1 VS T2), which revealed a region of low density of hemorrhagic focus in the right parietal lobe (Figure 1), coupled with a hyperdense area that showed thrombosis of the left transverse and superior sagittal sinus (Figures 2a, 2b). Enzyme-linked immunosorbent assay (ELISA) was positive for anti-platelet factor 4 (PF4) antibody the next day, and screening for antiphospholipid antibodies, direct antiglobulin, ADAMST13 with its antibodies was negative.

**Figure 3:**
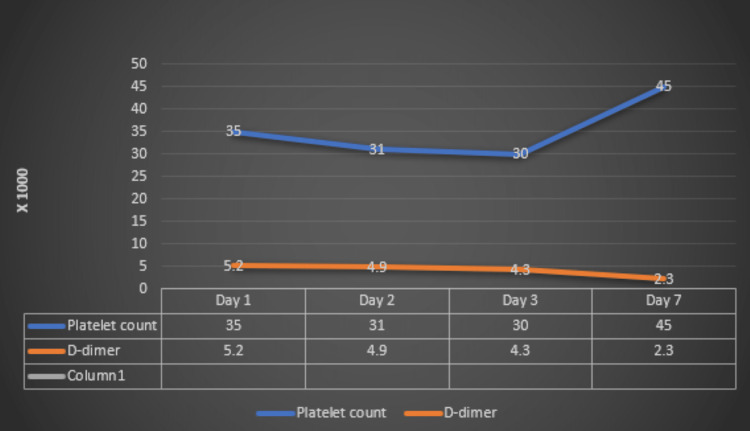
Patient's platelet count and D-dimer levels during her hospital stay.

She was diagnosed with vaccine-related TTS based on serological and imaging modalities. She was managed with high-dose dexamethasone, weight-adjusted intravenous immunoglobulin (IVIG), and anticoagulation (apixaban). She was observed closely, and her clinical symptoms started improving over 24 hours with an adequate platelet increase. She was self-ambulatory and nearly resolved neurological symptoms at discharge after 11 days. On follow-up two weeks later, she remained on apixaban 2.5mg twice daily with a normal hemogram. Her platelet count and D-dimer levels during the hospital stay are shown in Figure 3.

## Discussion

VITT and heparin-induced thrombocytopenia are similar because platelet-activating autoantibodies are formed in both disorders due to exposure to the COVID vaccine and heparin products, respectively, leading to consumptive thrombocytopenia and thrombotic complications [5]. Safe and effective vaccination against COVID-19 remains the mainstay of control of coronavirus. Most vaccines are well-tolerated and have shown promising efficacy and immunogenicity; however, vaccine-associated side effects and adverse events have also been reported [7]. Headache, myalgia, malaise, and injection site reactions are commonly reported side effects of the COVID-19 vaccine. Vaccine-associated adverse events can be classified into gastrointestinal, cardiovascular, hematological, neurological, and immunological [7]. Thrombosis, coagulation disorders, thrombocytopenia, abnormalities in fibrinogen levels, D-dimer count, or partial thromboplastin time are hematological complications reported in many patients [8]. VITT, a life-threatening emergency, has been described as a complication of adenoviral COVID-19 vaccines in the literature, although not widely reported. We have tabulated the reported cases of VITT following AstraZeneca COVID-19 vaccination in Table 2 [4-6,9-11]. The mechanism of VITT needs to be clearly defined, and it has been reported that COVID-19 vaccine-associated VITT presents 5-48 days following vaccination. Postvaccination antibodies against platelet antigens trigger massive platelet activation against PF4, leading to ITT. Activation of platelets against PF4 has also been proposed as a mechanism of VITT. Cross-reactivity between PF4, platelets, and vaccine leads to an immune-mediated response and formation of immune complexes, a potential contributing factor in the pathogenesis of VITT, leading to platelet activation and thrombosis [4,5,12].

**Table 2:** Reported cases of VITT after AstraZeneca COVID-19 vaccine. F: female, M: male, COVID-19: coronavirus disease 2019.

Author et al.	Age/Sex	Presenting complain	COVID-19 vaccine type	First presentation after vaccination (days)	Platelet count (150,000-350,000/mm3)	Imaging	Management
Butler-Manuel et al. [4]	48/F	Worsening headache, generalized fatigue	AstraZeneca	11	109,000	Thrombosis of right transverse and sigmoid sinus	Steroids, immunoglobulins
Mehta et al. [5]	32/M	Headache, left-sided hemiplegia	AstraZeneca	9	30,000	Thrombosis of superior sagittal sinus	No treatment given
Franchini et al. [6]	50/M	Worsening headache, Altered sensorium	AstraZeneca	7	15,000	Intraparenchymal hemorrhage, thrombosis of left transverse and sigmoid sinus	Neurosurgery, plasmapheresis
Dutta et al. [9]	51/M	Headache, vomiting	AstraZeneca	6	160,000	Thrombosis of superior sagittal and transverse sinus	Anticoagulation
Aladdin et al. [10]	36/F	Convulsions, left sided-body weakness	AstraZeneca	14	94,000	Superior sagittal thrombosis	Anticoagulation
Choi et al. [11]	33/F	Headache, vomiting	AstraZeneca	9	14,000	Thrombosis of left and right transverse sinus	Mechanical thrombectomy

Most patients diagnosed with VITT are young and female, without any pre-existing thrombotic risk factors or heparin administration history. VITT is managed with high-dose systemic glucocorticoids, non-heparin therapeutic anticoagulation, and IVIG. All heparin flushes must be avoided to prevent an increased risk of bleeding until VITT is ruled out. Platelet transfusions are recommended if the platelet count is below 30,000/mm^3^ and there is active bleeding. The fibrinogen level must be corrected via cryoprecipitate to maintain a more than 1.5g/L level. Eventually, all the patients are advised to keep anticoagulation for three months with regular follow-up [3,8,12].

Headache following COVID-19 vaccination is a common complication and usually resolves the following day. The recurrent and persistent headaches may raise clinical suspicion of VITT. In our patient, thrombosis was not detected by initial imaging performed on the first day, but on the following day, with marked thrombocytopenia, imaging finally revealed thrombosis of the right sagittal sinus. Hence, single imaging may be insufficient in patients with persistent or recurrent headaches following COVID-19 vaccination to rule out VITT. We assume that recurrent or persistent headaches after vaccination might represent a state of ongoing thrombosis in cerebral sinuses or veins. Ikenberg et al. reported a similar case in a young female who presented with a headache seven days following the COVID-19 vaccination. Her initial imaging was unremarkable. Her repeat magnetic resonance imaging revealed extensive thrombosis in cerebral veins three days later, and she was diagnosed with VITT. After commencing IVIG and anticoagulation, her symptoms resolved [13].

## Conclusions

Although the risk of VITT following the COVID-19 vaccine appears to be low, early diagnosis and management can be lifesaving. Our case highlights the importance of sequential imaging in patients who complain of persistent or recurrent headaches to identify the thrombosis when initial imaging is unremarkable to prevent severe morbidity and mortality. In these cases, a high index of suspicion could lead to an early diagnosis and aggressive intervention, which may help to prevent severe morbidity or mortality.
